# iPSC-Derived Pancreatic Progenitors Lacking FOXA2 Reveal Alterations in miRNA Expression Targeting Key Pancreatic Genes

**DOI:** 10.1007/s12015-023-10515-3

**Published:** 2023-02-07

**Authors:** Noura Aldous, Ahmed K. Elsayed, Nehad M. Alajez, Essam M. Abdelalim

**Affiliations:** 1 College of Health and Life Sciences, Hamad Bin Khalifa University (HBKU), Qatar Foundation (QF), Doha, Qatar; 2 Diabetes Research Center (DRC), Qatar Biomedical Research Institute (QBRI), Hamad Bin Khalifa University (HBKU), Qatar Foundation (QF), PO Box 34110, Doha, Qatar; 3 Translational Cancer and Immunity Center (TCIC), Qatar Biomedical Research Institute (QBRI), Hamad Bin Khalifa University (HBKU), Qatar Foundation (QF), PO Box 34110, Doha, Qatar

**Keywords:** Pancreatic development, Endocrine pancreas, Transcription factors, β-cells, miRNA-seq, RNA-Seq

## Abstract

Recently, we reported that forkhead box A2 (FOXA2) is required for the development of human pancreatic α- and β-cells. However, whether miRNAs play a role in regulating pancreatic genes during pancreatic development in the absence of FOXA2 expression is largely unknown. Here, we aimed to capture the dysregulated miRNAs and to identify their pancreatic-specific gene targets in pancreatic progenitors (PPs) derived from wild-type induced pluripotent stem cells (WT-iPSCs) and from iPSCs lacking FOXA2 (*FOXA2*^–/–^iPSCs). To identify differentially expressed miRNAs (DEmiRs), and genes (DEGs), two different *FOXA2*^–/–^iPSC lines were differentiated into PPs. *FOXA2*^–/–^ PPs showed a significant reduction in the expression of the main PP transcription factors (TFs) in comparison to WT-PPs. RNA sequencing analysis demonstrated significant reduction in the mRNA expression of genes involved in the development and function of exocrine and endocrine pancreas. Furthermore, miRNA profiling identified 107 downregulated and 111 upregulated DEmiRs in *FOXA2*^–/–^ PPs compared to WT-PPs. Target prediction analysis between DEmiRs and DEGs identified 92 upregulated miRNAs, predicted to target 1498 downregulated genes in *FOXA2*^–/–^ PPs. Several important pancreatic TFs essential for pancreatic development were targeted by multiple DEmiRs. Selected DEmiRs and DEGs were further validated using RT-qPCR. Our findings revealed that FOXA2 expression is crucial for pancreatic development through regulating the expression of pancreatic endocrine and exocrine genes targeted by a set of miRNAs at the pancreatic progenitor stage. These data provide novel insights of the effect of *FOXA2* deficiency on miRNA-mRNA regulatory networks controlling pancreatic development and differentiation.

**Graphical Abstract:**

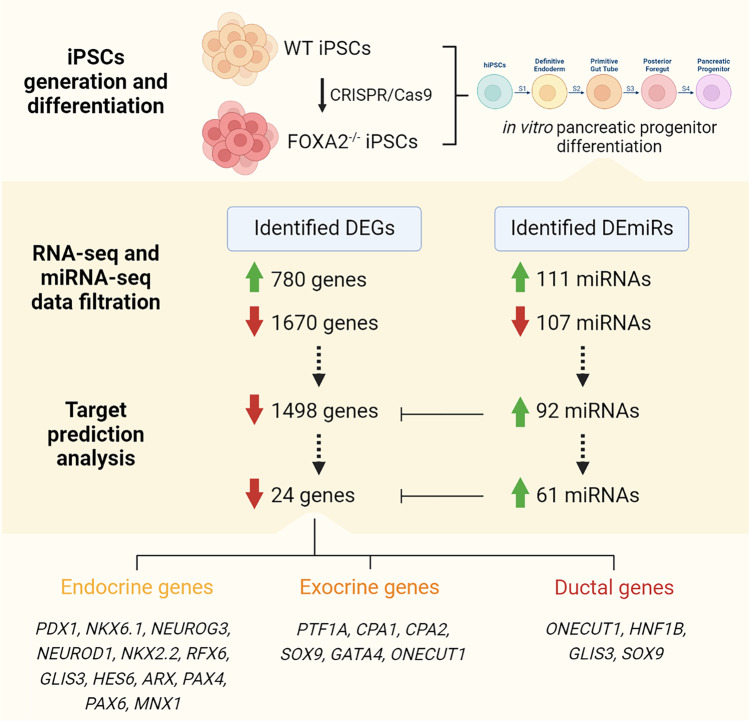

**Supplementary Information:**

The online version contains supplementary material available at 10.1007/s12015-023-10515-3.

## Introduction

Forkhead Box A2 (FOXA2) is one of the earliest transcription factors (TFs) expressed during pancreatic development and remains to be expressed in all types of pancreatic cells [1]. During human pancreatic organogenesis, FOXA2 starts to be expressed at 4 weeks of gestation and continues onwards [2–4]. Previous studies demonstrated that FOXA2 controls the expression of several TFs and genes involved in pancreatic endocrine cell fate and β-cell functionality [5, 6]. Using human pluripotent stem cells (hPSCs), we and others reported that FOXA2 plays very important roles during human pancreatic and hepatic development [7–9]. A recent study reported that heterozygous missense variants in *FOXA2* can lead to monogenic diabetes [10]. Another study showed that in humans, risk alleles of type 2 diabetes (T2D) are associated with FOXA2-bound enhancers [11]. These findings indicate the contribution of *FOXA2* defects in diabetes development and its important role during pancreatic endocrine differentiation.

Contributions of microRNAs (miRNAs) to gene regulation during pancreatic β-cell development is well-established, where they play important roles in enhancing the generation of stem cell-derived pancreatic islets and diabetes pathogenesis [12, 13]. miRNAs are known to suppress target mRNAs [14, 15]; however, it has been suggested that miRNAs may also enhance gene expression [16]. Single miRNA can suppress several mRNA targets, and multiple miRNAs may have influence on a specific pathway [15, 17]. Previous studies reported that several miRNAs play crucial roles in regulating the development and function of pancreatic β-cells [12, 13, 18] and glucose homeostasis [19]. Examples of those miRNAs are miR-26, miR-24, and miR-148 [20], miR-375 [13], miR-21 [21], miR-30d [22, 23], let-7 [24], miR-34a and miR-34c [25], miR-9 [26], and miR-7 [27]. Furthermore, miRNAs have been found to be involved in maintaining β-cell identity [28, 29]. Dysregulated expression of several miRNAs has been reported to be associated with diabetes development [28, 30]. In mouse pancreatic progenitors (PPs), a specific deletion of Dicer1 enzyme, which is universally required for the functional miRNA maturation, led to decreased pancreatic endocrine cell numbers [31]. Furthermore, disruption of Dicer1 in pancreatic β-cells impairs insulin biosynthesis [20].

Recent progress in human induced PSC (hiPSC) technology has paved the way for many essential applications that could be used for disease modeling, targeted therapy, drug screening, and precision medicine. Therefore, here, we take advantage of our recently established FOXA2 knockout hiPSC (*FOXA2*^–/–^iPSC) model to identify the alterations in the miRNA and mRNA profiles in PPs lacking *FOXA2* to understand the miRNA-mRNA regulatory networks regulating pancreatic development. Our results showed that loss of *FOXA2* leads to the upregulation of numerous miRNAs targeting key PP genes involved in pancreatic exocrine and endocrine development.

## Materials and Methods

### Culture and Differentiation of iPSCs Into Pancreatic Progenitors

iPSC lines (Ctr1-iPSCs and Ctr2-iPSCs) generated and fully characterized in our laboratory were used as we previously reported [32]. *FOXA2* knockout iPSCs from Ctr1-and Ctr2-iPSCs were generated using CRISPR/Cas9 as we recently reported [5]. Both wild-type (WT) and *FOXA2*^*–/–*^ iPSCs were cultured and maintained using Stemflex media (ThermoFisher Scientific) on Matrigel-coated plates (Corning). iPSC lines were differentiated into PPs using our established protocol (Supplementary Table 1) [33–35].

### Immunocytochemistry

Immunostaining was performed on differentiated iPSCs as previously reported [32, 36]. Cells were washed once with PBS then 4% paraformaldehyde (PFA) was added on the cells for 20 min and placed on a shaker at room temperature. The cells were then washed with tris-buffered saline + 0.5% Tween 20 (TBST) thrice in a 10-minute interval on a shaker. Cells were then permeabilized for 15 min at room temperature using phosphate buffered saline (PBS) + 0.5% Triton X-100 (PBST) twice, later blocked overnight with 6% Bovine Serum Albumin (BSA) in PBST at 4^o^C. Afterwards, guinea pig anti-PDX1 (1:500, ab47308, Abcam) and mouse anti-NKX6.1 (1:2000, F55A12-C, DSHB) primary antibodies diluted in 3% BSA in PBST were added to the cells and incubated overnight at 4^o^C. Cells were washed three times with TBST and then Alexa Fluor secondary antibodies (ThermoFisher Scientific) diluted in PBS (1:500) were added for 1 h at room temperature then washed again three times using TBST. Cell nuclei were stained for two minutes with Hoechst 33,258 (DAPI) diluted 1:5000 in PBS (Life Technologies, USA). After washing three times with PBS, images were captured using inverted fluorescence microscope (Olympus).

### Western Blotting

Total protein was extracted from one well of a 6-well plate using RIPA lysis buffer with protease inhibitor (ThermoFisher Scientific). Measurement of protein concentration was done using Pierce BCA kit (ThermoFisher Scientific). 20 µg of total protein were separated by SDS-PAGE and transferred onto PVDF membranes. Membranes were blocked with 10% skimmed milk in TBST then incubated with rabbit anti-FOXA2 (1:4000, #3143, Cell Signaling) and mouse anti-β-actin (1:10,000, sc-47,778, Santa Cruz) primary antibodies overnight at 4^o^C. Membranes were washed with TBST then horseradish peroxidase-conjugated secondary antibodies (Jackson Immunoresearch) diluted in TBST (1:10,000) were added for 1 h at room temperature then washed again using TBST. Membranes were developed using SuperSignal West Pico Chemiluminescent substrate (Pierce, Loughborough, UK) then visualized using iBright™ CL 1000 Imaging System (Invitrogen).

### RNA Extraction and RT-qPCR Analysis

1 × 10^6^ cells were collected using 700 µL of TRIzol Reagent (Life Technologies) then total RNA extraction was performed using Direct-zol™ RNA Miniprep (Zymo Research, USA). For mRNA, cDNA was synthesized from 1 µg of RNA using SuperScript™ IV First-Strand Synthesis System following manufacturer protocol (ThermoFisher Scientific, USA). RT-qPCR was performed using GoTaq qPCR SYBR Green Master Mix (Promega, USA) run in triplicates. Average Ct values were normalized to the WT samples for each gene tested. GAPDH was used as an endogenous control (primer details are listed in Supplementary Table 2).

For miRNA RT-qPCR validation, 5 ng/µL of total RNA was reverse transcribed using miRCURY LNA RT Kit (QIAGEN, Cat. No. 339,340) then diluted 1:30 using RNase-free water. Relative miRNA expression levels were determined using miRCURY LNA SYBR® Green PCR Kit (QIAGEN, Cat. No. 339,345) and miRCURY LNA miRNA PCR Assay. hsa-miR-122-5p (Assay ID: YP00205664), hsa-miR-184 (Assay ID: YP00204601), hsa-miR-9-5p (Assay ID: YP00204513), hsa-miR-371a-3p (Assay ID: YP00204299), hsa-miR-371a-5p (Assay ID: YP00204493), hsa-miR-194-5p (Assay ID: YP00204080), hsa-miR-885-5p (Assay ID: YP00204473), hsa-miR-373-3p (Assay ID: YP00204604), and hsa-miR-493-3p (Assay ID: YP00204557), were used. Relative miRNA expression was calculated using –ΔΔCT. SNORD48 was used as endogenous control for miRNA expression.

### Differential Gene Expression Analysis

Following manufacturer’s protocol, NEBNext Poly(A) mRNA Magnetic Isolation Kit (NEB, E7490) was used for capturing mRNA using 1 µg of total RNA. Generation of RNA-sequencing (RNA-seq) libraries was done using NEBNext Ultra Directional RNA Library Prep Kit (NEB, E7420L) and libraries were sequenced using Illumina Hiseq 4000 system. Raw data were converted to FASTQ files using Illumina BCL2Fastq Conversion Software v2.20 while running quality controls in parallel. Pair-end FASTQ files were subsequently aligned to the GRCh38 reference genome using built-in module and default settings in CLC genomics workbench v21.0.5. Normalized expression data (TPM (transcripts per million)) mapped reads were sequentially imported into the AltAnalyze v.2.1.3 software for differential expression analysis as we described before. (10.3390/cancers13215350) For identifying DEGs, genes with log2 fold change (FC) > 1 and <-1 with a *P*-value < 0.05 were considered. Gene ontology (GO) and Kyoto Encyclopedia of Genes and Genomes (KEGG) pathways analyses were performed using the Database for Annotation, Visualization and Integrated Discovery (DAVID) [37].

### Differential miRNA Expression and Potential Target Analysis

miRNA expression profiling was conducted on differentiated and collected PP total RNA samples from WT and *FOXA2*^*–/–*^ iPSCs. From the extracted total RNA, ~ 100 ng was used for miRNA library preparation following the manufacturer’s instructions of the library kit (E7560S, New England BioLabs Inc., USA). The amplified cDNA constructs were purified using the Monarch PCR purification kit (Biolabs, New England). MicroRNA analysis was carried out in CLC genomics workbench 20.0 using built-in small RNA analysis workflow. miRNA count reads were normalized using the TMM (trimmed mean of M values) normalization method and log2 CPM (Counts per Million) values were subsequently subjected to differential analysis. A log2 FC > 1 with a *P*-value < 0.05 was used as a cutoff to determine the differentially expressed miRNA in *FOXA2*^*–/–*^ iPSCs versus WT-iPSCs. Pathway analysis and the microRNA target filter were employed to identify potential miRNA–mRNA networks using Ingenuity Pathway Analysis (IPA) software (QIAGEN, Germany).

### Statistical Analysis

At least three biological replicates were used in most of the experiments, otherwise technical replicates were used for statistical analyses. Statistical analysis was performed using unpaired two-tailed student’s t-test by Prism 8 software. Data are represented as mean ± standard deviation (SD).

## Results

### Identification of Differentially Expressed Genes in iPSC-Derived Pancreatic Progenitors Lacking *FOXA2*

**Fig. 1:**
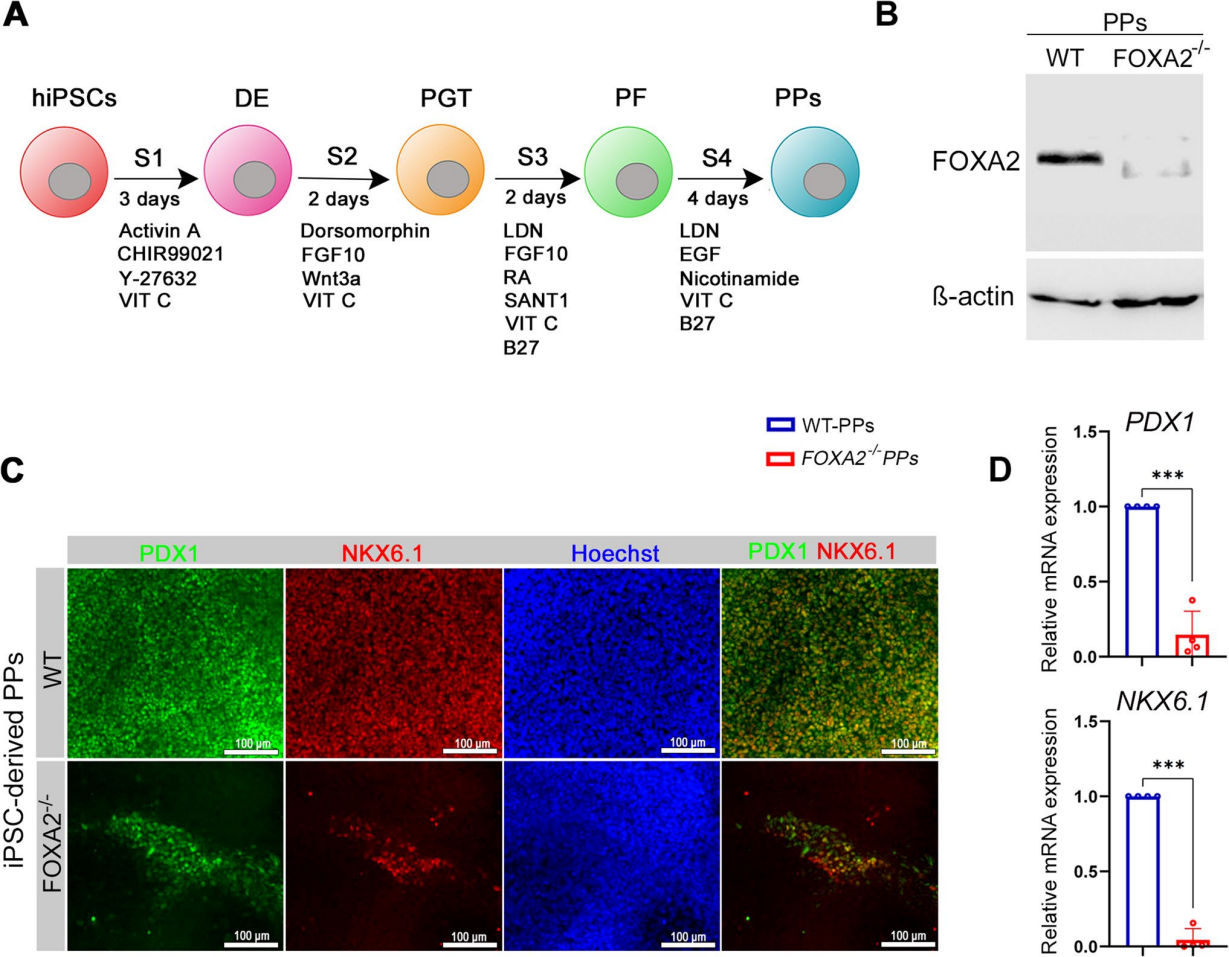
Effect of *FOXA2* loss on pancreatic progenitor (PP) differentiation.** A** Schematic representation of iPSC differentiation protocol into pancreatic progenitors (PPs). **B** Western blot analysis confirming the absence of FOXA2 expression in PPs derived from *FOXA2*^*–/–*^ iPSCs. Immunofluorescence (**C**) and RT-qPCR (**D**) showing significant reduction in the expression of PDX1 and NKX6.1 in *FOXA2*^*–/–*^ PPs. Scale bars = 100 μm. Data are represented as mean ± SD; ***p < 0.001 (*n* = 4)

To investigate the effects of *FOXA2* loss on mRNA and miRNA expression in PPs, we used two CRISPR/Cas9-generated *FOXA2*^*–/–*^ iPSC lines with their isogenic controls (WT-iPSCs) as we recently reported [7]. Generated iPSCs were differentiated into PPs that co-express PDX1 and NKX6.1 using our established differentiation protocol (Fig. 1A) [33]. The expression levels of FOXA2 in WT- and *FOXA2*^*–/–*^ PPs were validated at protein level using Western blotting where there was a clear absence of FOXA2 band in *FOXA2*^*–/–*^ PPs (Fig. 1B). At PP stage, *FOXA2*^*–/–*^ PPs showed a significant decrease in the expression levels of the two key progenitor TFs, PDX1 and NKX6.1, as indicated by immunocytochemistry and RT-qPCR (Fig. 1C, D) which is concordant with our previously reported data [7].

**Fig. 2:**
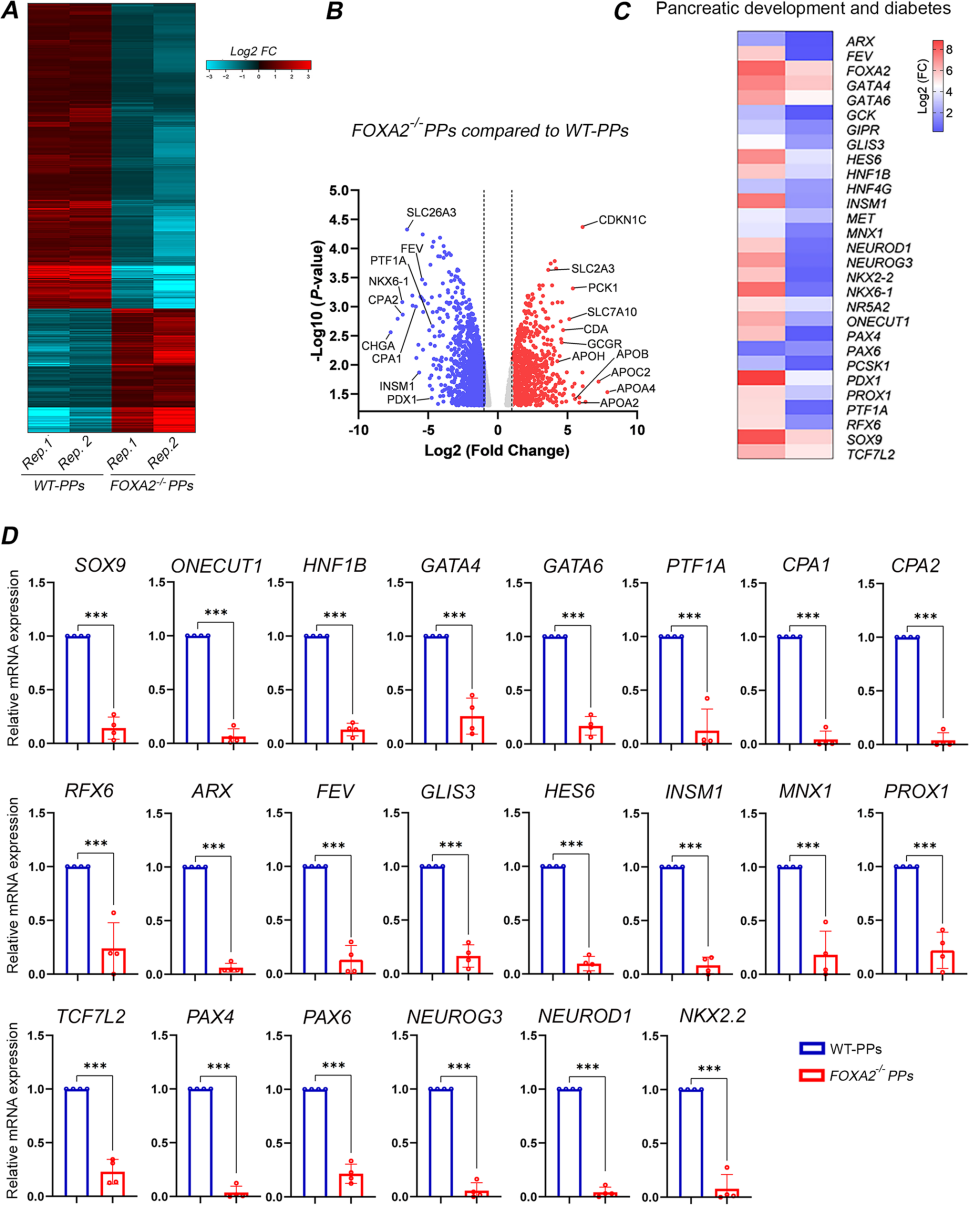
Transcriptomic changes in pancreatic progenitors (PPs) derived from *FOXA2*^***–/–***^iPSCs.** A** Heatmap of differentially expressed genes (DEGs) in WT-PPs and FOXA2^–/–^ PPs generated from two biological replicates. **B** Volcano plots showing the DEGs. The blue dots indicate downregulated, and the red dots indicate upregulated mRNAs in *FOXA2*^*–/–*^ PPs compared to WT-PPs. **C** Heatmap of downregulated DEGs in *FOXA2*^*–/–*^ PPs, associated with pancreatic development and diabetes. **E** RT-qPCR analysis for validation of selected DEGs from RNA-seq results (*n* = 4). Data are represented as mean ± SD; ***p < 0.001

To identify differentially expressed genes (DEGs) in iPSC-derived PPs from WT and FOXA2 lacking iPSCs, we performed RNA-Seq analysis. The transcriptome analysis revealed 780 significantly upregulated (Log2 FC > 1.0, *p* < 0.05) and 1670 significantly downregulated (Log2 FC < − 1.0, *p* < 0.05) DEGs in *FOXA2*^*–/–*^ PPs compared with WT-PPs (Fig. 2A, B). Selected important downregulated DEGs in *FOXA2*^*–/–*^ PPs are listed in Supplementary Table 3. Principle component analysis (PCA) of the WT- and *FOXA2*^*–/–*^ PPs clustered the data into two distinct groups indicating the difference in transcriptomic profiles between the two groups (Supplementary Fig. 1A). GO and KEGG pathways enrichment analyses on downregulated DEGs revealed genes association with pancreatic development and diabetes (Fig. 2C and Supplementary Fig. 1B). As for upregulated DEGs, pathways’ enrichments were mostly associated with cholesterol and lipid metabolism, complement and coagulation cascades, glucose metabolism, and other metabolic pathways (Supplementary Fig. 1C). To validate RNA-Seq data, RT-qPCR was performed on several key pancreatic DEGs (Fig. 2D). Confirming the RNA-seq results, the mRNA expression of pancreatic and endocrine progenitor TFs including *SOX9, ONECUT1, HNF1B, GATA4, GATA6, PTF1A, RFX6, ARX, GLIS3, HES6, INSM1, MNX1, PROX1, TCF7L2, PAX4, PAX6, NEUROG3, NEUROD1, NKX2.2*, and *FEV* were significantly downregulated in PPs derived from *FOXA2*^*–/–*^ iPSCs (Fig. 2D). Furthermore, *CPA1* and *CPA2*, enzymes associated with exocrine pancreas, were significantly downregulated (Fig. 2D). Those DEGs were also validated in the PPs derived from the other *FOXA2*^*−/−*^iPSC line and its WT controls (Supplementary Fig. 2). On the other hand, absence of FOXA2 was associated with upregulation of several genes, which were mostly associated with cholesterol and lipid metabolism as indicated by the increased expression of apolipoprotein (APO) genes (*APOA4*, *APOC2*, *APOA2*, *APOB*, *APOA1*, *APOH*, *APOM*, *APOE*, *APOC1*, *APOA5*, and *APOL6*), *ABCG5*, and *ABCG8* (Supplementary Table 4).

### Identification of Differentially Expressed miRNAs in iPSC-Derived Pancreatic Progenitors Lacking *FOXA2*

**Fig. 3:**
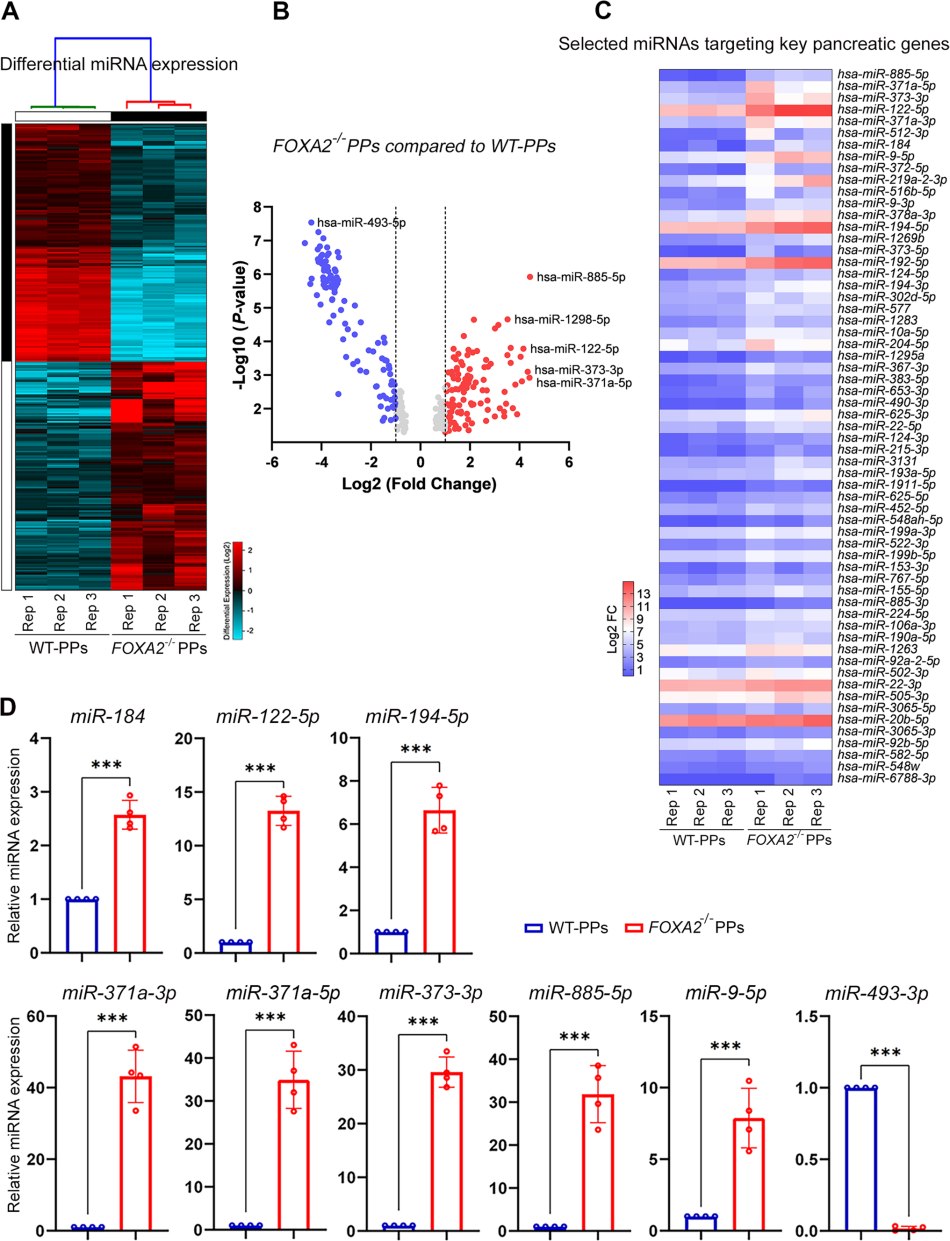
miRNA profiling of pancreatic progenitors (PPs) derived from *FOXA2*^***–/–***^ iPSCs and WT-PPs.** A** Heatmap of downregulated and upregulated DEmiRs in WT-PPs and *FOXA2*^*–/–*^ PPs generated from three biological replicates (*n* = 3). **B** Volcano plots showing the DEmiRs. The blue dots indicate downregulated, and the red dots indicate upregulated miRNAs in *FOXA2*^*–/–*^ PPs compared to WT-PPs. **C** A heatmap showing selected upregulated DEmiRs targeting key pancreatic genes. **D** Validation of selected DEmiRs from miRNA-Seq results. Data are represented as mean ± SD; ***p < 0.001 (*n* = 4)

To identify the altered miRNA expression profile, we performed miRNA-Seq from the same collected RNA samples from *FOXA2*^*–/–*^ iPSCs and WT-iPSCs derived PPs. The miRNA-Seq analysis identified 111 significantly upregulated (Log2 FC > 1.0, *p* < 0.05) and 107 significantly downregulated (Log2 FC < − 1.0, *p* < 0.05) differentially expressed miRNAs (DEmiRs) in *FOXA2*^*–/–*^ PPs compared to WT-PPs (Fig. 3A, B). PCA of the WT- and *FOXA2*^*–/–*^ PPs clustered the data into two distinct groups indicating the difference in miRNA expression profiles between the two groups (Supplementary Fig. 3). Upregulated and downregulated miRNAs (Supplementary Tables 5 and 6) may play roles in pancreatic islet development and function. Since our data revealed significant suppression of β-cell development in the absence of FOXA2, in this study, we focused on upregulated miRNAs in the absence of FOXA2. The top upregulated DEmiRs (Log2 FC > 1.0, *p* < 0.05) are listed in Supplementary Table 5. Many of those DEmiRs are involved in regulating the expression of key β-cell development genes (Fig. 3C). RT-qPCR was used to validate the expression of selected DEmiRs. RT-qPCR analysis showed significant upregulation of miR-194-5p, miR-371a-3p, miR-371a-5p, miR-122-5p, miR-184, miR-9-5p, miR-885-5p, and miR-373-3p and significant downregulation of miR-493-3p (Fig. 3D).

### Pancreatic Genes Are Potential Targets of Upregulated miRNAs in *FOXA2*^–/–^ Pancreatic Progenitors

**Fig. 4:**
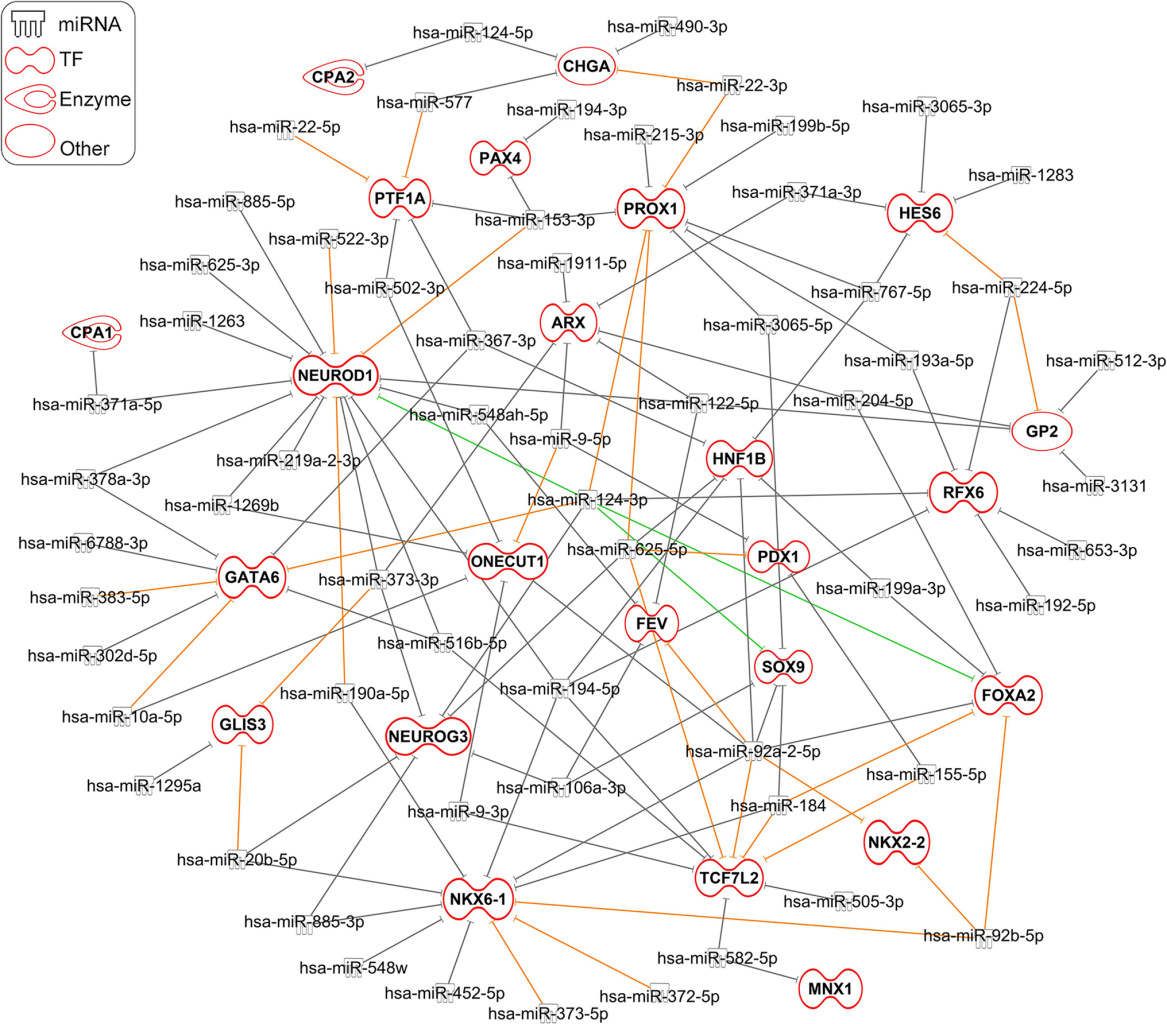
Network of upregulated differentially expressed miRNAs (DEmiRs) and predicted target pancreatic genes in pancreatic progenitors. The Ingenuity Pathway Analysis (IPA) tool was used to construct the regulatory network between the upregulated miRNAs and the predicted downregulated gene targets. Experimentally observed miRNA targets are indicated by the green lines, highly predicted targets are indicated by the orange lines, and moderately predicted targets are represented by the grey lines. Predicted miRNA targets are classified as transcription factors (TFs), enzymes, or others (see key legends)

To further understand the contribution of the upregulated miRNAs in regulating pancreatic development in the absence of FOXA2 expression, we integrated our miRNA and mRNA data using IPA to identify miRNA targets. Target prediction for the significantly upregulated DEmiRs (Log2 FC > 1.0, *p* < 0.05) identified 92 miRNAs predicted to target 1498 significantly downregulated DEGs (Log2 FC < − 1.0, *p* < 0.05) in *FOXA2*^*–/–*^ PPs. Here, we focused on selected targets from the DEGs known to play essential role in pancreatic development. Target prediction analysis identified several of the downregulated pancreatic DEGs as potential targets for several of the upregulated DEmiRs in PPs lacking *FOXA2* (Fig. 4; Table 1). FOXA2 was the predicted target for 6 different DEmiRs including hsa-miR-184, hsa-miR-204-5p, hsa-miR-124-3p, hsa-miR-199a-3p, hsa-miR-92a-2-5p, and hsa-miR-92b-5p. The main PP TF, PDX1, was a predicted target for hsa-miR-9-5p, hsa-miR-625-5p, and hsa-miR-155-5p, while NKX6.1 was a predicted target for 11 upregulated DEmiRs: hsa-miR-184, hsa-miR-372-5p, hsa-miR-194-5p, hsa-miR-373-5p, hsa-miR-452-5p, hsa-miR-885-3p, hsa-miR-190a-5p, hsa-miR-92a-2-5p, hsa-miR-20b-5p, hsa-miR-92b-5p, and hsa-miR-548w. NEUROD1 was the predicted target for 15 DEmiRs: hsa-miR-885-5p, hsa-miR-371a-5p, hsa-miR-373-3p, hsa-miR-122-5p, hsa-miR-219a-2-3p, hsa-miR-516b-5p, hsa-miR-378a-3p, hsa-miR-194-5p, hsa-miR-1269b, hsa-miR-625-3p, hsa-miR-124-3p, hsa-miR-548ah-5p, hsa-miR-522-3p, hsa-miR-153-3p, hsa-miR-190a-5p, and hsa-miR-1263. ONECUT1 and GATA6 were the predicted targets for 9 and 7 upregulated DEmiRs, respectively, while CPA1, CPA2, and MNX1 were targeted by only one miRNA. Interestingly, hsa-miR-124-3p targeted multiple important pancreatic TFs including FOXA2, NEUROG3, NEUROD1, GATA6, SOX9, INSM1, and RFX6 (Fig. 4; Table 1). Also, hsa-miR-291a-3p was found to have several target genes including NEUROG3, GLIS3, ARX, and NEUROD1. In addition, we also observed miRNA targets overlapped where a single miRNA was predicted to target multiple essential pancreatic gene markers (Fig. 4; Table 1). Using TargetScan Human and miRecords databases in IPA analysis, the confidence of DEmiRs target genes was assessed and classified either experimentally observed or predicted with moderate or high levels of confidence. Upregulated miR-124-3p in *FOXA2*^–/–^PPs was experimentally validated to be targeting FOXA2, NEUROD1, and SOX9. In highly DEmiRs, 22 miRNAs were highly predicted to be targeting multiple key PP markers that are essential for pancreatic islet differentiation. These data highlighted empirical roles for the upregulated in miRNAs in regulating the expression of their *bone fide* gene targets in the context of pancreatic cell development. Functional roles of the identified mRNA-miRNA networks remain to be validated experimentally.

**Table 1:** Upregulated miRNAs and their predicted target genes (selected) downregulated in FOXA2−/− PPs compared with WT-iPSCs

Upregulated miRNA	Log2 FC	*P*-value	Predicted target gene	Log2 FC	*P*-value
hsa-miR-124-5p	2.582	0.00058	CHGA	-7.697	0.00276
hsa-miR-577	2.158	2.26E-05			
hsa-miR-490-3p	1.866	0.00241			
hsa-miR-22-3p	1.131	0.000837			
hsa-miR-184	3.828	0.00193	NKX6-1	-6.865	0.000836
hsa-miR-372-5p	3.457	0.00708			
hsa-miR-194-5p	2.833	0.000466			
hsa-miR-373-5p	2.718	0.0108			
hsa-miR-452-5p	1.519	0.00133			
hsa-miR-885-3p	1.301	0.00918			
hsa-miR-190a-5p	1.261	0.0069			
hsa-miR-92a-2-5p	1.166	0.0132			
hsa-miR-20b-5p	1.089	0.0174			
hsa-miR-92b-5p	1.07	0.0305			
hsa-miR-548w	1.046	0.00743			
hsa-miR-124-5p	2.582	0.00058	CPA2	-6.826	0.00137
hsa-miR-373-3p	4.331	0.000796	NEUROG3	-6.12	0.000963
hsa-miR-124-3p	1.674	0.00226			
hsa-miR-625-5p	1.526	0.00267			
hsa-miR-885-3p	1.301	0.00918			
hsa-miR-106a-3p	1.263	0.000799			
hsa-miR-20b-5p	1.089	0.0174			
hsa-miR-371a-5p	4.406	0.0012	CPA1	-5.879	0.000994
hsa-miR-194-3p	2.508	0.00282	PAX4	-5.55	0.000672
hsa-miR-153-3p	1.423	0.0311			
hsa-miR-122-5p	4.157	0.000164	FEV	-5.444	0.000341
hsa-miR-548ah-5p	1.512	0.0127			
hsa-miR-106a-3p	1.263	0.000799			
hsa-miR-92a-2-5p	1.166	0.0132			
hsa-miR-122-5p	4.157	0.000164	GP2	-5.359	0.00123
hsa-miR-512-3p	3.904	0.0146			
hsa-miR-204-5p	2.079	0.0272			
hsa-miR-3131	1.566	0.00787			
hsa-miR-224-5p	1.288	0.000988			
hsa-miR-92a-2-5p	1.166	0.0132	NKX2-2	-5.313	0.000787
hsa-miR-92b-5p	1.07	0.0305			
hsa-miR-885-5p	4.424	1.2E-06	NEUROD1	-4.838	0.00438
hsa-miR-371a-5p	4.406	0.0012			
hsa-miR-373-3p	4.331	0.000796			
hsa-miR-122-5p	4.157	0.000164			
hsa-miR-219a-2-3p	3.374	0.0124			
hsa-miR-516b-5p	3.2	0.00969			
hsa-miR-378a-3p	3.01	4.09E-05			
hsa-miR-194-5p	2.833	0.000466			
hsa-miR-1269b	2.73	0.000559			
hsa-miR-625-3p	1.748	0.00323			
hsa-miR-124-3p	1.674	0.00226			
hsa-miR-548ah-5p	1.512	0.0127			
hsa-miR-522-3p	1.462	0.000723			
hsa-miR-153-3p	1.423	0.0311			
hsa-miR-190a-5p	1.261	0.0069			
hsa-miR-1263	1.252	0.0121			
hsa-miR-9-5p	3.512	0.000254	PDX1	-4.748	0.0369
hsa-miR-625-5p	1.526	0.00267			
hsa-miR-155-5p	1.357	0.0258			
hsa-miR-577	2.158	2.26E-05	PTF1A	-4.67	0.00217
hsa-miR-367-3p	1.934	0.00172			
hsa-miR-22-5p	1.746	0.0026			
hsa-miR-153-3p	1.423	0.0311			
hsa-miR-502-3p	1.15	0.0454			
hsa-miR-9-5p	3.512	0.000254	ONECUT1	-4.174	0.00159
hsa-miR-9-3p	3.144	3.23E-05			
hsa-miR-1269b	2.73	0.000559			
hsa-miR-10a-5p	2.113	0.000213			
hsa-miR-92a-2-5p	1.166	0.0132			
hsa-miR-502-3p	1.15	0.0454			
hsa-miR-194-5p	2.833	0.000466	RFX6	-3.661	0.0258
hsa-miR-192-5p	2.648	0.000352			
hsa-miR-653-3p	1.874	0.0141			
hsa-miR-124-3p	1.674	0.00226			
hsa-miR-193a-5p	1.543	0.0392			
hsa-miR-224-5p	1.288	0.000988			
hsa-miR-371a-3p	4.042	0.000285	HES6	-3.305	0.0128
hsa-miR-1283	2.134	0.00224			
hsa-miR-767-5p	1.421	0.0026			
hsa-miR-224-5p	1.288	0.000988			
hsa-miR-3065-3p	1.087	0.00201			
hsa-miR-184	3.828	0.00193	SOX9	-2.872	0.00414
hsa-miR-124-3p	1.674	0.00226			
hsa-miR-106a-3p	1.263	0.000799			
hsa-miR-92a-2-5p	1.166	0.0132			
hsa-miR-3065-5p	1.093	0.0273			
hsa-miR-582-5p	1.059	0.00297	MNX1	-2.574	0.0469
hsa-miR-373-3p	4.331	0.000796	GLIS3	-2.467	0.006
hsa-miR-1295a	1.99	0.000662			
hsa-miR-20b-5p	1.089	0.0174			
hsa-miR-184	3.828	0.0305	FOXA2	-2.449	0.00981
hsa-miR-204-5p	2.079	0.0272			
hsa-miR-124-3p	1.674	0.00226			
hsa-miR-199a-3p	1.473	0.000612			
hsa-miR-92a-2-5p	1.166	0.0132			
hsa-miR-92b-5p	1.07	0.0305			
hsa-miR-124-3p	1.674	0.00226	PROX1	-2.316	0.0358
hsa-miR-215-3p	1.636	0.0122			
hsa-miR-193a-5p	1.543	0.0392			
hsa-miR-625-5p	1.526	0.00267			
hsa-miR-199b-5p	1.432	0.000336			
hsa-miR-153-3p	1.423	0.0311			
hsa-miR-767-5p	1.421	0.0026			
hsa-miR-22-3p	1.131	0.000837			
hsa-miR-3065-5p	1.093	0.0273			
hsa-miR-194-5p	2.833	0.000466	HNF1B	-2.285	0.00222
hsa-miR-367-3p	1.934	0.00172			
hsa-miR-625-5p	1.526	0.00267			
hsa-miR-199a-3p	1.473	0.000612			
hsa-miR-767-5p	1.421	0.0026			
hsa-miR-92a-2-5p	1.166	0.0132			
hsa-miR-373-3p	4.331	0.000796	ARX	-1.914	0.00541
hsa-miR-122-5p	4.157	0.000164			
hsa-miR-371a-3p	4.042	0.000285			
hsa-miR-9-5p	3.512	0.000254			
hsa-miR-204-5p	2.079	0.0272			
hsa-miR-1911-5p	1.528	0.000232			
hsa-miR-516b-5p	3.2	0.00969	GATA6	-1.901	0.00538
hsa-miR-378a-3p	3.01	4.09E-05			
hsa-miR-302d-5p	2.229	0.00165			
hsa-miR-10a-5p	2.113	0.000213			
hsa-miR-367-3p	1.934	0.00172			
hsa-miR-383-5p	1.884	0.00123			
hsa-miR-124-3p	1.674	0.00226			
hsa-miR-6788-3p	1.004	0.05			
hsa-miR-184	3.828	0.00193	TCF7L2	-1.177	0.0282
hsa-miR-516b-5p	3.2	0.00969			
hsa-miR-9-3p	3.144	3.23E-05			
hsa-miR-194-5p	2.833	0.000466			
hsa-miR-625-5p	1.526	0.00267			
hsa-miR-155-5p	1.357	0.0258			
hsa-miR-92a-2-5p	1.166	0.0132			
hsa-miR-505-3p	1.099	0.0225			
hsa-miR-582-5p	1.059	0.00297			

## Discussion

FOXA2 is an important TF that starts to be expressed at a very early stage of pancreatic development, where the first expression is detected at the definitive endoderm stage and continues in all stages. Our recent study showed that loss of *FOXA2* during pancreatic differentiation of iPSCs prevents the formation of α- and β-cells [7]. However, there are currently no data available on the effects of FOXA2 deficiency on the expression pattern of miRNAs and their specific targets in PPs. Here, we provide evidence that FOXA2 deficiency is associated with significant alterations in the expression levels of miRNAs targeting key pancreatic genes at PP stage. The alterations in miRNA expression in PP derived from iPSCs lacking FOXA2 may reflect an impairment in pancreatic differentiation. A direct role for FOXA2 in regulating the expression of selected miRNAs warrants further investigation.

PPs are characterized by the expression of several TFs and genes involved in directing the PPs into different types of pancreatic cells (endocrine, exocrine, and ductal cells). Our recent report showed that loss of one *FOXA2* allele in iPSCs generated from a patient with *FOXA2* haploinsufficiency significantly reduced the expression of pancreatic TFs involved in the development of endocrine pancreas [7]. In agreement with these findings, our RNA-Seq and RT-qPCR results showed loss of *FOXA2* to significantly downregulate the expression of key endocrine-associated genes, such as *PDX1, NKX6.1, NEUROG3, NEUROD1, NKX2.2, RFX6, GLIS3, HES6, ARX, PAX4, PAX6, MNX1, GATA6, FEV, INSM1, TCF7L2, GP2*, and *CHGA*, which were targeted by several upregulated miRNAs in PPs lacking *FOXA2. ARX* and *PAX4* are known to be essential for the formation of pancreatic α-cells and β-cells, respectively [38, 39]. Furthermore, the downregulated genes associated with exocrine and ductal cell specification such as *PTF1A, CPA1, CPA2, SOX9, GATA4*, and *ONECUT1* were also targeted by several upregulated miRNAs, indicating that FOXA2 is not only essential for pancreatic endocrine development, but also plays an important role in pancreatic exocrine and ductal development. Many of those downregulated genes are associated with diabetes and pancreatic development. These findings indicate that lack of *FOXA2* negatively impacted the iPSC differentiation into exocrine and endocrine pancreas through downregulating the expression of essential pancreatic developmental genes.

miRNAs are known to play essential roles in post-transcriptional regulation through targeting mRNAs [40, 41]. The role of miRNAs in regulating pancreatic β-cell development and function has been previously reported [12]. However, limited studies have tackled the role of miRNAs in regulating PP development. In the current study, we noticed that most DEmiRs had several predicted targets in PPs. On the other hand, most of key pancreatic targets were predicted targets for at least two DEmiRs. For example, we found that miR-184 expression level was upregulated and among its predicted targets is NKX6.1, which is a key TF in pancreatic endocrine development and later becomes restricted to pancreatic β-cells [42, 43]. It has been reported that miR-184 participates in regulating β-cell expansion and negatively correlates with insulin biosynthesis and secretion [44–46]. These results suggest the correlation between miR-184 expression and pancreatic β-cell generation and function. Here, we identified multiple miRNAs targeting early important PP markers such as SOX9, which was predicted to be targeted by hsa-miR-3065-5p, hsa-miR-106a-3p, hsa-miR-92a-2-5p, hsa-miR-184, and hsa-miR-124-3p. Studies have showed that the expression of SOX9 during early pancreatic development is important for the generation of all three pancreatic cell lineages (endocrine, exocrine, and ductal) [1]. Inactivation of Sox9 leads to pancreatic hypoplasia in mouse models, where Sox9 has been found to be essential for survival and proliferation of PPs [47]. Hence, the identified DEmiRs targeting SOX9 might act as potential regulators in PPs development. HNF1B is another important TF was predicted to be targeted by hsa-miR-199a-3p, hsa-miR-767-5p, hsa-miR-367-3p, hsa-miR-625-5p, hsa-miR-194-5p, and hsa-miR-92a-2-5p. HNF1B is important during pancreas development [48] where its mutation causes monogenic diabetes in humans (reviewed in [49]), and functions upstream of SOX9 and NEUROG3 [50, 51] reflecting its late regulatory role during PP development.

We have also identified an upregulation of miR-9-5p in our *FOXA2*^*–/–*^ PPs, targeting PDX1, ONECUT1, and ARX, which were significantly downregulated. Previous studies have linked the upregulation of miR-9 cluster of miRNAs with glucose-stimulated insulin secretion impairments [26]. This cluster has also been identified as a regulator of insulin exocytosis and secretion machinery through modulating Sirt1 expression [52]. A previous study showed that miR-9 targets Onecut2 and decreases its mRNA expression in pancreatic β-cells, which subsequently leads to an increase in Onecut2 downstream target, granuphilin (a negative regulator of the insulin exocytosis) [26]. miR-124a (i.e., a precursor for miR-124-3p/5p) is expressed in human islets and has been reported to be associated with T2D. It has been found that miR-124a represses important target genes involved in pancreatic β-cell function and insulin secretion [53], including *Foxa2* and *Pdx1* [54]. Although our analysis did not show NKX6.1 as a predicted target for miR-124-5p, it has recently been reported that it induces pancreatic β-cell differentiation by regulating NKX6.1 expression [55]. In this study, miR-124-3p from the same miRNA cluster, was significantly increased in *FOXA2*^*–/–*^ PPs and its predicted targets were the downregulated pancreatic TFs, NEUROG3, PROX1, RFX6, GATA6 as well as NEUROD1, SOX9, and FOXA2, which were experimentally validated in previous research [53, 56]. Our results showed miR-92a-2-5p among the upregulated DEmiRs that targets eight downregulated key pancreatic TFs including FOXA2, NKX6-1, FEV, NKX2-2, ONECUT1, SOX9, HNF1B, and TCF7L2. A recent study found that miR-92a-2-5p regulates insulin production and pancreatic β-cell apoptosis [57, 58]. Our results showed increased expression of miR-577 upon *FOXA2* loss. Previous studies showed that miR-577 inhibits pancreatic β-cell activity and survival by targeting FGF21, which promotes β-cell function and survival through AKT signaling pathway [59, 60]. miR-204 was found to be associated with the endocrine part of pancreatic islets and insulin regulation [61, 62]. miR-15a-5p was also found to regulate insulin production by suppressing UCP-2 gene expression, a mitochondrial anion carrier that reduces oxidative stress [63], resulting in more insulin biosynthesis [64]. On the other hand, upregulation of miR-146a/b has been found to increase cytokine-induced β-cell apoptosis [65]. From these results, we speculate that the lack of FOXA2 at PP stage can cause alterations of several miRNAs important for pancreatic β-cell development and function from an early stage of pancreas development before reaching the mature β-cell stage, causing the cells to follow a different trajectory from the normal mono-hormonal functional β-cells. Taken together, these results show that the lack of *FOXA2* alters PP differentiation, at least in part, through upregulating the expression of several miRNAs, which have a biological impact on pancreas development. Furthermore, the data suggest that the pancreatic development is regulated through a complex miRNA network targeting important pancreatic genes at PP stage. Further experiments are needed to investigate the role of the top DEmiRs identified in this study during pancreatic development.

miRNAs have been also identified as epigenetic modifiers that regulate gene expression levels without targeting its mRNA sequence but by targeting important enzymes including DNA methyltransferases (DNMTs), histone methyltransferases (HMT), and histone deacetylases (HDACs) [66, 67]. In addition, miRNAs are posed to epigenetic modification and regulation such as DNA methylation and RNA/histone modifications. The interchangeable relationship between miRNAs and epigenetic modifications forms the bases of miRNA-epigenetic feedback loop that can affect cellular processes [68], physiological functions and disease conditions [69]. Recently, a study has discovered that FOXA2 physically interacts with ten-eleven-translocation methylcytosine dioxygenase 1 (TET1) in which β-cell specification is significantly hindered upon TET1 loss [70]. This lays a good example of TF crosstalk with epigenetic regulators in regulating pancreatic β-cell differentiation and specification. Our miRNA-seq data identified several DEmiRs which have been previously associated with epigenetic modifications in different tissue samples [71, 72]. We predict that the lack of FOXA2 does not only affect miRNAs regulating other genes, but also affects miRNAs regulating epigenetic modifications that can directly affect histone modification for accessing DNA for transcription. It was previously found that alterations in circulating miRNA expression occur in diabetic patients in which they can be even be used as biomarkers for diabetes prediction and progression [73, 74]. miRNAs can also be used as biomarkers for pancreatic cancer progression and prognosis [75]. Furthermore, alterations in gene regulation by miRNA can be the cause some forms of pancreatic cancers as some miRNAs can act as oncogenes and are associated with poor disease prognosis [76]. Another important aspect of miRNAs is that they can serve as potential therapeutic agents for regenerative medicine [77, 78]. Recent advances in research have led to the development of miRNA delivery systems to regulate gene expression [77]. Therefore, our identified DEmiRs may serve as potential novel biomarkers or therapeutic modulators for diabetes or pancreatic cancer diseases. However, further functional validation is required to provide a proof-of-concept for the link between identified miRNAs and diseases.

In conclusion, we showed that FOXA2 loss led to dysregulation of several miRNAs and mRNAs expressed in iPSC-derived PPs. Our findings demonstrated that FOXA2 is not only crucial for endocrine islet development, but also it is essential for exocrine pancreas development. Integrating miRNA and mRNA profiling results revealed that the potential targets of DEmiRs identified in this study are known to play an essential role in pancreatic development and function. These data provide proof of the regulatory relationship between pancreatic TFs and miRNAs in controlling the expression of main pancreatic differentiation drivers during pancreatic islet differentiation. Also, the data presented here would serve as the platform for future studies focusing on understanding the function of identified DEmiRs. In addition, further understanding of miRNA-mRNA and miRNA-epigenetic feedback loop would help in identifying potential novel therapeutic strategies and targets that are not limited to *FOXA2* mutations but include cancer and regenerative medicine.

## Supplementary Information

Below is the link to the electronic supplementary material.

ESM 1 (DOCX 14.2 KB)

ESM 2 (DOCX 16.2 KB)

ESM 3 (DOCX 16.1 KB)

ESM 4 (DOCX 16.0 KB)

ESM 5 (DOCX 24.4 KB)

ESM 6 (DOCX 25.0 KB)

ESM 7 (PDF 513 KB)

ESM 8 (PDF 446 KB)

ESM 9 (PDF 189 KB)

## Data Availability

The data that support the findings of this study are available from the corresponding author upon reasonable request.
