# Supplementary material for: iPSC-Derived Pancreatic Progenitors Lacking FOXA2 Reveal Alterations in miRNA Expression Targeting Key Pancreatic Genes
Source: Stem Cell Rev Rep. 2023 Feb 7;19(4):1082–97. doi: 10.1007/s12015-023-10515-3 (PMC10185633; doi:10.1007/s12015-023-10515-3)
Supplement: Supplementary file 1 — (DOCX 14.2 KB) [file 12015_2023_10515_MOESM1_ESM.docx]

**Supplementary Table 1.** Pancreatic progenitor differentiation protocol *in vitro*.

| **Stage of Differentiation** | **Media** | **Final Cytokine Concentration** |
| --- | --- | --- |
| Stage 1:  Definitive Endoderm (Day 1) | MCDB131  1% Pen/Strep  1% L-Glutamine  10 mM Glucose  0.5% BSA  1.5 g/L NaHCO_3_ | 100 ng/mL Activin A  0.25 mM Vitamin C  2 µM CHIR99021  1 mM Rock Inhibitor |
| Stage 1:  Definitive Endoderm (Days 2-3) | MCDB131  1% Pen/Strep  1% L-Glutamine  10 mM Glucose  0.5% BSA  1.5 g/L NaHCO_3_ | 100 ng/mL Activin A  0.25 mM Vitamin C |
| Stage 2: Primitive Gut Tube (Days 4-6) | MCDB131  1% Pen/Strep  1% L-Glutamine  10 mM Glucose  0.5% BSA  1.5 g/L NaHCO_3_ | 3 ng/mL Wnt3a  0.75 µM Dorsomorphin  0.25 mM Vitamin C  50 ng/mL FGF10 |
| Stage 3: Posterior Foregut (Days 7-8) | DMEM  1% Pen/Strep  1% L-Glutamine  4.5 g/L D-Glucose  110 mg/L Sodium Pyruvate | 1% B27  200 nM LDN  0.25 mM Vitamin C  50 ng/mL FGF10  2 µM RA  0.25 µM SANT-1 |
| Stage 4: Pancreatic Progenitor (Days 9-12) | DMEM  1% Pen/Strep  1% L-Glutamine  4.5 g/L D-Glucose  110 mg/L Sodium Pyruvate | 1% B27  200 nM LDN  100 ng/mL EGF  0.25 mM Vitamin C  10 mM Nicotinamide |
