# Supplementary material for: iPSC-Derived Pancreatic Progenitors Lacking FOXA2 Reveal Alterations in miRNA Expression Targeting Key Pancreatic Genes
Source: Stem Cell Rev Rep. 2023 Feb 7;19(4):1082–97. doi: 10.1007/s12015-023-10515-3 (PMC10185633; doi:10.1007/s12015-023-10515-3)
Supplement: Supplementary file 2 — (DOCX 16.2 KB) [file 12015_2023_10515_MOESM2_ESM.docx]

**Supplementary Table 2.** List of primers used for RT-qPCR analysis

| **Gene** | **Forward** | **Reverse** |
| --- | --- | --- |
| *FOXA2* | GGGAGCGGTGAAGATGGA | TCATGTTGCTCACGGAGGAGTA |
| *PDX1* | CGTCCAGCTGCCTTTCCCAT | CCGTGAGATGTACTTGTTGAATAGGA |
| *NKX6.1* | GGGCTCGTTTGGCCTATTCGTT | CCACTTGGTCCGGCGGTTCT |
| *GATA4* | GCCTCCTCTGCCTGGTAAT | CAGTCCCATCAGCGTGTAAA |
| *GATA6* | AAGCGCGTGCCTTCATCA | TCATAGCAAGTGGTCTGGGC |
| *NEUROD1* | GCCCCAGGGTTATGAGACTAT | GAGAACTGAGACACTCGTCTGT |
| *NEUROG3* | GGCTGTGGGTGCTAAGGGTAAG | CAGGGAGAAGCAGAAGGAACAA |
| *NKX2.2* | AAACCATGTCACGCGCTCA | GGCGTTGTACTGCATGTGCT |
| *ONECUT1* | GGACCTCAAGATAGCAGGTTTAT | CAGAATGCAGGTGAGCTAAGT |
| *HNF1B* | ACACACCTCCCATCCTCAAG | CATTTTAGCAGCCCTCCAAG |
| *INSM1* | TTTGTCTCGTGGTTGGAAGC | CCAAAACAACCCGTACGCTA |
| *PAX4* | AGCAGAGGCACTGGAGAAAGAGTT | CAGCTGCATTTCCCACTTGAGCTT |
| *PAX6* | GCGGAAGCTGCAAAGAAATAG | GGGCAAACACATCTGGATAATG |
| *PROX1* | AAAGTCAAATGTACTCCGCAAGC | CTGGGAAATTATGGTTGCTCCT |
| *RFX6* | GTCGATGCATGGCTTGGACT | TGGGCCATAGCTAGACGGTG |
| *ARX* | CTGCTGAAACGCAAACAGAGGC | CTCGGTCAAGTCCAGCCTCATG |
| *CPA1* | ACTACGCCACCTACCACACC | GGTGTTGCCAATCTGGATCT |
| *CPA2* | ATCTTCCTCCTGCCAGTCAC | CACACCAACACAGAGGCTTC |
| *FEV* | GCCTCTCCAAACTCAACCTC | CAAGCTGGGACTGGGGTAG |
| *GLIS3* | GTGAAGGCACATTCTTCCAAAGA | ACAGAGCTCCATCCAGACCTGC |
| *GLUT2* | ATGAACTGCCCACAATCTCATA | GGACCAGAGCATGGTGATTAG |
| *MNX1* | CGAGACCCAGGTGAAGATTT | CTTCTGTTTCTCCGCTTCCT |
| *PTF1A* | CCAGAAGGTCATCATCTGCC | AGAGAGTGTCCTGCTAGGGG |
| *SOX9* | GACTACACCGACCACCAGAACTCC | GTCTGCGGGATGGAAGGGA |
| *TCF7L2* | CGTAGACCCCAAAACAGGAA | TCCTGTCGTGATTGGGTACA |
| *APOA2* | GTTCGGAGACAGGCAAAGGA | TCAAAGTAAGACTTGGCCTCGG |
| *HES6* | AGCCCCTGGTGGAGAAGA | CAGCACTCCGGCGTTCTC |
| *GAPDH* | ACGACCACTTTGTCAAGCTCATTTC | GCAGTGAGGGTCTCTCTCTTCCTCT |
