# Supplementary material for: iPSC-Derived Pancreatic Progenitors Lacking FOXA2 Reveal Alterations in miRNA Expression Targeting Key Pancreatic Genes
Source: Stem Cell Rev Rep. 2023 Feb 7;19(4):1082–97. doi: 10.1007/s12015-023-10515-3 (PMC10185633; doi:10.1007/s12015-023-10515-3)
Supplement: Supplementary file 3 — (DOCX 16.1 KB) [file 12015_2023_10515_MOESM3_ESM.docx]

Supplementary Table 3. Selected key downregulated DEGs in *FOXA2^–/–^* PPs compared with WT-PPs (P < 0.05)

| **Gene ID** | **Log2 FC** | ***P*-value** |
| --- | --- | --- |
| *CHGA* | -7.697 | 0.00276 |
| *NKX6-1* | -6.865 | 0.000836 |
| *CPA2* | -6.826 | 0.00137 |
| *NEUROG3* | -6.120 | 0.000963 |
| *CPA1* | -5.879 | 0.000994 |
| *INSM1* | -5.653 | 0.013488 |
| *PAX4* | -5.550 | 0.000672 |
| *FEV* | -5.444 | 0.000341 |
| *GP2* | -5.359 | 0.001233 |
| *NKX2-2* | -5.313 | 0.000787 |
| *DKK2* | -5.019 | 0.000881 |
| *NEUROD1* | -4.838 | 0.004375 |
| *PDX1* | -4.748 | 0.036873 |
| *PTF1A* | -4.670 | 0.002167 |
| *ONECUT1* | -4.174 | 0.00159 |
| *DLK1* | -3.954 | 0.000397 |
| *RFX6* | -3.661 | 0.025842 |
| *HES6* | -3.305 | 0.012777 |
| *IGF2* | -2.938 | 0.012407 |
| *SOX9* | -2.871 | 0.004139 |
| *MNX1* | -2.574 | 0.046898 |
| *GLIS3* | -2.467 | 0.006005 |
| *GCK* | -2.449 | 0.001482 |
| *FOXA2* | -2.448 | 0.009809 |
| *ADCY1* | -2.381 | 0.011858 |
| *PROX1* | -2.316 | 0.035846 |
| *HNF1B* | -2.284 | 0.002219 |
| *DLL4* | -2.252 | 0.006545 |
| *CHGB* | -2.212 | 0.008774 |
| *ADCY5* | -2.132 | 0.001001 |
| *PCSK1* | -2.126 | 0.013956 |
| *CD24* | -2.064 | 0.030191 |
| *ARX* | -1.914 | 0.005407 |
| *GATA6* | -1.901 | 0.005377 |
| *NR5A2* | -1.622 | 0.00322 |
| *GATA4* | -1.499 | 0.010756 |
| *CPA4* | -1.398 | 0.04519 |
| *TCF7L2* | -1.177 | 0.028179 |
| *TCF7* | -1.098 | 0.008595 |
| *HNF4G* | -1.000 | 0.008878 |
