# Supplementary material for: iPSC-Derived Pancreatic Progenitors Lacking FOXA2 Reveal Alterations in miRNA Expression Targeting Key Pancreatic Genes
Source: Stem Cell Rev Rep. 2023 Feb 7;19(4):1082–97. doi: 10.1007/s12015-023-10515-3 (PMC10185633; doi:10.1007/s12015-023-10515-3)
Supplement: Supplementary file 4 — (DOCX 16.0 KB) [file 12015_2023_10515_MOESM4_ESM.docx]

Supplementary Table 4. Selected key upregulated DEGs in *FOXA2^–/–^* PPs compared with WT-PPs (P < 0.05)

| **Gene ID** | **Log2 FC** | ***P*-value** |
| --- | --- | --- |
| *APOA4* | 7.866 | 0.029359 |
| *APOC2* | 7.226 | 0.019385 |
| *APOA2* | 5.853 | 0.044592 |
| *APOB* | 5.424 | 0.033082 |
| *PCK1* | 5.381 | 0.000487 |
| *APOA1* | 4.903 | 0.042478 |
| *DPP4* | 4.735 | 0.03107 |
| *GCGR* | 4.579 | 0.004129 |
| *ARG1* | 4.444 | 0.007052 |
| *APOH* | 4.269 | 0.008667 |
| *HKDC1* | 3.909 | 0.034437 |
| *SLC2A3* | 3.615 | 0.000233 |
| *ABCC2* | 3.465 | 0.012955 |
| *GLUT-2* | 3.326 | 0.009322 |
| *ALDOB* | 3.306 | 0.03441 |
| *APOM* | 3.276 | 0.036989 |
| *ABCG5* | 3.238 | 0.001113 |
| *APOE* | 3.230 | 0.006505 |
| *APOC1* | 2.902 | 0.048535 |
| *CEBPA* | 2.671 | 0.027329 |
| *GCKR* | 2.619 | 0.010879 |
| *APOA5* | 2.558 | 0.039732 |
| *ANXA1* | 2.433 | 0.001357 |
| *APOL6* | 2.246 | 0.015672 |
| *APOC4-APOC2* | 2.213 | 0.010197 |
| *EGF* | 2.198 | 0.009222 |
| *ABCG8* | 1.931 | 0.025729 |
| *DPPA3* | 1.891 | 0.00256 |
| *WNT5A* | 1.683 | 0.002881 |
| *ABCA1* | 1.605 | 0.008066 |
| *BMP2* | 1.424 | 0.016743 |
| *WNT11* | 1.238 | 0.027865 |
| *SLC16A1* | 1.208 | 0.007184 |
| *DPPA2* | 1.180 | 0.02574 |
| *ABCA7* | 1.158 | 0.018869 |
| *SLC3A2* | 1.155 | 0.035476 |
| *KLF8* | 1.145 | 0.00701 |
| *WNT10B* | 1.136 | 0.023083 |
