# Supplementary material for: iPSC-Derived Pancreatic Progenitors Lacking FOXA2 Reveal Alterations in miRNA Expression Targeting Key Pancreatic Genes
Source: Stem Cell Rev Rep. 2023 Feb 7;19(4):1082–97. doi: 10.1007/s12015-023-10515-3 (PMC10185633; doi:10.1007/s12015-023-10515-3)
Supplement: Supplementary file 5 — (DOCX 24.4 KB) [file 12015_2023_10515_MOESM5_ESM.docx]

**Supplementary Table 5.** Top upregulated differentially expressed miRNAs with identified predicted targets in *FOXA2^–/–^* PPs compared with WT-PPs (*P* < 0.05).

| **miRNA ID** | **miRNA symbol** | **Log2 FC** | ***P*-value** |
| --- | --- | --- | --- |
| hsa-miR-885-5p | miR-885-5p | 4.424 | 1.20E-06 |
| hsa-miR-371a-5p | miR-293-5p | 4.406 | 0.001200969 |
| hsa-miR-373-3p | miR-291a-3p | 4.331 | 0.000796461 |
| hsa-miR-122-5p | miR-122-5p | 4.157 | 0.000164395 |
| hsa-miR-371a-3p | miR-292-3p | 4.042 | 0.000284868 |
| hsa-miR-512-3p | miR-512-3p | 3.904 | 0.014567303 |
| hsa-miR-184 | miR-184 | 3.828 | 0.001926378 |
| hsa-miR-1323 | miR-5480-3p | 3.728 | 0.009592192 |
| hsa-miR-146b-5p | miR-146a-5p | 3.643 | 0.000433577 |
| hsa-miR-1298-5p | miR-1298-5p | 3.517 | 2.21E-05 |
| hsa-miR-9-5p | miR-9-5p | 3.512 | 0.000253828 |
| hsa-miR-372-5p | miR-295-5p | 3.457 | 0.007079625 |
| hsa-miR-122-3p | miR-122-3p | 3.395 | 0.003161678 |
| hsa-miR-526b-5p | miR-526b-5p | 3.380 | 0.016883808 |
| hsa-miR-219a-2-3p | miR-219a-2-3p | 3.374 | 0.012401494 |
| hsa-miR-516b-5p | miR-516b-5p | 3.200 | 0.009688099 |
| hsa-miR-9-3p | miR-9-3p | 3.144 | 3.23E-05 |
| hsa-miR-378a-3p | miR-378a-3p | 3.010 | 4.09E-05 |
| hsa-miR-517a-3p | miR-517a-3p | 3.002 | 0.016931234 |
| hsa-miR-194-5p | miR-194-5p | 2.833 | 0.000465739 |
| hsa-miR-1269b | miR-1269a | 2.730 | 0.000559448 |
| hsa-miR-373-5p | miR-292b-5p | 2.718 | 0.01075876 |
| hsa-miR-519c-5p | miR-526a-5p | 2.700 | 0.005636564 |
| hsa-miR-219a-5p | miR-219a-5p | 2.679 | 0.002850581 |
| hsa-miR-518b | miR-518a-3p | 2.652 | 0.023007304 |
| hsa-miR-192-5p | miR-192-5p | 2.648 | 0.000351701 |
| hsa-miR-124-5p | miR-124-5p | 2.582 | 0.000580461 |
| hsa-miR-194-3p | miR-194-3p | 2.508 | 0.002816545 |
| hsa-miR-195-5p | miR-16-5p | 2.427 | 0.006927796 |
| hsa-miR-516a-5p | miR-516a-5p | 2.285 | 0.000192206 |
| hsa-miR-302d-5p | miR-302b-5p | 2.229 | 0.00165116 |
| hsa-miR-577 | miR-577 | 2.158 | 2.26E-05 |
| hsa-miR-1283 | miR-1283 | 2.134 | 0.002242322 |
| hsa-miR-10a-5p | miR-10a-5p | 2.113 | 0.000212521 |
| hsa-miR-129-2-3p | miR-129-1-3p | 2.079 | 0.007667521 |
| hsa-miR-204-5p | miR-204-5p | 2.079 | 0.027154423 |
| hsa-miR-302c-5p | miR-302c-5p | 2.015 | 0.001128167 |
| hsa-miR-1295a | miR-1295a | 1.990 | 0.000662066 |
| hsa-miR-944 | miR-944 | 1.979 | 0.001327774 |
| hsa-miR-498-5p | miR-498-5p | 1.941 | 0.043735406 |
| hsa-miR-367-3p | miR-92a-3p | 1.934 | 0.001715884 |
| hsa-miR-192-3p | miR-192-3p | 1.925 | 0.020757908 |
| hsa-miR-205-5p | miR-205-5p | 1.899 | 0.001857864 |
| hsa-miR-489-3p | miR-489-3p | 1.894 | 0.000255687 |
| hsa-miR-383-5p | miR-383-5p | 1.884 | 0.001230608 |
| hsa-miR-653-3p | miR-653-3p | 1.874 | 0.014094857 |
| hsa-miR-490-3p | miR-490-3p | 1.866 | 0.002414031 |
| hsa-miR-1264 | miR-1264 | 1.752 | 6.88E-05 |
| hsa-miR-625-3p | miR-625-3p | 1.748 | 0.00322729 |
| hsa-miR-22-5p | miR-22-5p | 1.746 | 0.002595339 |
| hsa-miR-378a-5p | miR-378a-5p | 1.741 | 0.001812999 |
| hsa-miR-302a-5p | miR-302a-5p | 1.738 | 0.005418316 |
| hsa-miR-520h | miR-520g-3p | 1.734 | 0.002688641 |
| hsa-miR-124-3p | miR-124-3p | 1.674 | 0.002255128 |
| hsa-miR-187-3p | miR-187-3p | 1.639 | 0.01378953 |
| hsa-miR-215-3p | miR-215-3p | 1.636 | 0.012217931 |
| hsa-miR-3131 | miR-3131 | 1.566 | 0.007868782 |
| hsa-miR-193a-5p | miR-193a-5p | 1.543 | 0.039248061 |
| hsa-miR-1911-5p | miR-1911-5p | 1.528 | 0.000231575 |
| hsa-miR-625-5p | miR-625-5p | 1.526 | 0.002668252 |
| hsa-miR-452-5p | miR-452-5p | 1.519 | 0.001331973 |
| hsa-miR-548ah-5p | miR-3609 | 1.512 | 0.012730984 |
| hsa-miR-199a-3p | miR-199a-3p | 1.473 | 0.000611863 |
| hsa-miR-522-3p | miR-224-3p | 1.462 | 0.000723469 |
| hsa-miR-199b-5p | miR-199a-5p | 1.432 | 0.000336441 |
| hsa-miR-153-3p | miR-153-3p | 1.423 | 0.031146174 |
| hsa-miR-767-5p | miR-767 | 1.421 | 0.002602945 |
| hsa-miR-876-3p | miR-876-3p | 1.388 | 0.039516119 |
| hsa-miR-155-5p | miR-155-5p | 1.357 | 0.02583773 |
| hsa-miR-488-5p | miR-488-5p | 1.340 | 0.000295051 |
| hsa-miR-676-3p | miR-676-3p | 1.339 | 0.000165374 |
| hsa-miR-885-3p | miR-885-3p | 1.301 | 0.009177268 |
| hsa-miR-18b-3p | miR-18b-3p | 1.267 | 0.002787424 |
| hsa-miR-106a-3p | miR-106a-3p | 1.263 | 0.000799062 |
| hsa-miR-190a-5p | miR-190a-5p | 1.261 | 0.006895849 |
| hsa-miR-1263 | miR-1263 | 1.252 | 0.012143616 |
| hsa-miR-105-5p | miR-105-5p | 1.212 | 0.017514658 |
| hsa-miR-92a-2-5p | miR-92a-2-5p | 1.166 | 0.013161311 |
| hsa-miR-502-3p | miR-501-3p | 1.150 | 0.045362099 |
| hsa-miR-126-5p | miR-126a-5p | 1.146 | 0.030216424 |
| hsa-miR-22-3p | miR-22-3p | 1.131 | 0.000836853 |
| hsa-miR-505-3p | miR-505-3p | 1.099 | 0.022459038 |
| hsa-miR-3065-5p | miR-3065-5p | 1.093 | 0.02728358 |
| hsa-miR-20b-5p | miR-17-5p | 1.089 | 0.017394372 |
| hsa-miR-3065-3p | miR-3065-3p | 1.087 | 0.002010938 |
| hsa-miR-20b-3p | miR-20b-3p | 1.082 | 0.007363739 |
| hsa-miR-92b-5p | miR-92b-5p | 1.070 | 0.030506256 |
| hsa-miR-582-5p | miR-582-5p | 1.059 | 0.002970376 |
| hsa-miR-548w | miR-548h-5p | 1.046 | 0.007434575 |
| hsa-miR-548am-3p | miR-548ae-3p | 1.024 | 0.023135118 |
| hsa-miR-6788-3p | miR-6788-3p | 1.004 | 0.049989697 |
