# Supplementary material for: iPSC-Derived Pancreatic Progenitors Lacking FOXA2 Reveal Alterations in miRNA Expression Targeting Key Pancreatic Genes
Source: Stem Cell Rev Rep. 2023 Feb 7;19(4):1082–97. doi: 10.1007/s12015-023-10515-3 (PMC10185633; doi:10.1007/s12015-023-10515-3)
Supplement: Supplementary file 6 — (DOCX 25.0 KB) [file 12015_2023_10515_MOESM6_ESM.docx]

**Supplementary Table 6.** Top downregulated differentially expressed miRNAs with identified predicted targets in *FOXA2*^-/-^ PPs compared with WT-PPs (*P* < 0.05).

| **miRNA ID** | **miRNA Symbol** | **Log2 FC** | ***P*-value** |
| --- | --- | --- | --- |
| hsa-miR-493-3p | miR-493-3p | -4.667 | 0.000000119 |
| hsa-miR-412-5p | miR-412-5p | -4.453 | 0.00000195 |
| hsa-miR-493-5p | miR-493-5p | -4.413 | 2.91E-08 |
| hsa-miR-433-3p | miR-433-3p | -4.397 | 0.00000135 |
| hsa-miR-539-3p | miR-485-3p | -4.185 | 0.00000418 |
| hsa-miR-1185-1-3p | let-7f-2-3p | -4.153 | 0.000000182 |
| hsa-miR-543 | miR-543-3p | -4.141 | 0.000000358 |
| hsa-miR-495-3p | miR-495-3p | -4.123 | 5.57E-08 |
| hsa-miR-410-3p | miR-344d-3p | -4.116 | 0.000000429 |
| hsa-miR-381-3p | miR-381-3p | -4.065 | 0.000000148 |
| hsa-miR-889-3p | miR-889-3p | -4.035 | 0.00000796 |
| hsa-miR-323a-3p | miR-323-3p | -4.016 | 0.000000293 |
| hsa-miR-382-3p | miR-382-3p | -3.986 | 0.000000488 |
| hsa-miR-376a-3p | miR-376a-3p | -3.981 | 0.000000419 |
| hsa-miR-432-5p | miR-432 | -3.97 | 0.000000265 |
| hsa-miR-675-5p | miR-675-5p | -3.92 | 8.59E-08 |
| hsa-miR-370-3p | miR-370-3p | -3.919 | 0.00000154 |
| hsa-miR-654-3p | miR-654-3p | -3.909 | 0.00000206 |
| hsa-miR-487b-3p | miR-487b-3p | -3.899 | 0.000000786 |
| hsa-miR-154-3p | miR-154-3p | -3.889 | 0.000000674 |
| hsa-miR-431-3p | miR-431-3p | -3.87 | 0.000000565 |
| hsa-miR-127-5p | miR-127-5p | -3.852 | 0.00000243 |
| hsa-miR-154-5p | miR-154-5p | -3.825 | 0.000000409 |
| hsa-miR-377-5p | miR-377-5p | -3.813 | 0.00000249 |
| hsa-miR-411-5p | miR-411-5p | -3.784 | 0.00000162 |
| hsa-miR-494-3p | miR-494-3p | -3.778 | 0.000000276 |
| hsa-miR-654-5p | miR-541-3p | -3.77 | 0.000000159 |
| hsa-miR-409-5p | miR-409-5p | -3.748 | 0.0000006 |
| hsa-miR-136-3p | miR-136-3p | -3.738 | 0.000000798 |
| hsa-miR-127-3p | miR-127-3p | -3.722 | 0.000000817 |
| hsa-miR-382-5p | miR-382-5p | -3.707 | 0.000000241 |
| hsa-miR-758-3p | miR-758-3p | -3.703 | 0.00000138 |
| hsa-miR-337-3p | miR-337-3p | -3.684 | 0.0000269 |
| hsa-miR-409-3p | miR-409-3p | -3.671 | 0.00000136 |
| hsa-miR-431-5p | miR-431-5p | -3.631 | 0.00000237 |
| hsa-miR-136-5p | miR-136-5p | -3.626 | 0.00000109 |
| hsa-miR-370-5p | miR-370-5p | -3.59 | 0.00000134 |
| hsa-miR-675-3p | miR-675-3p | -3.569 | 0.00000196 |
| hsa-miR-369-5p | miR-369-5p | -3.568 | 0.00000582 |
| hsa-miR-541-5p | miR-541-5p | -3.554 | 0.00000215 |
| hsa-miR-299-5p | miR-299a-5p | -3.531 | 0.00000145 |
| hsa-miR-329-3p | miR-329-3p | -3.517 | 0.0000171 |
| hsa-miR-539-5p | miR-539-5p | -3.483 | 0.00000193 |
| hsa-miR-379-5p | miR-379-5p | -3.479 | 0.00000285 |
| hsa-miR-485-5p | miR-485-5p | -3.467 | 0.00000349 |
| hsa-miR-380-5p | miR-758-5p | -3.452 | 0.00000243 |
| hsa-miR-655-3p | miR-374c-5p | -3.431 | 0.000000816 |
| hsa-miR-369-3p | miR-369-3p | -3.408 | 0.00000219 |
| hsa-miR-376a-5p | miR-376a-5p | -3.38 | 0.00000124 |
| hsa-miR-411-3p | miR-411-3p | -3.357 | 0.00000188 |
| hsa-miR-299-3p | miR-299a-3p | -3.343 | 0.000000219 |
| hsa-miR-134-5p | miR-3118 | -3.321 | 0.00000151 |
| hsa-miR-335-3p | miR-335-3p | -3.316 | 0.0036334 |
| hsa-miR-377-3p | miR-377-3p | -3.294 | 0.000000315 |
| hsa-miR-487a-5p | miR-487a-5p | -3.21 | 0.0000116 |
| hsa-miR-1197 | miR-1197 | -3.125 | 0.000086 |
| hsa-miR-668-3p | miR-668-3p | -3.078 | 0.00000621 |
| hsa-miR-483-3p | miR-483-3p | -3.006 | 0.0002879 |
| hsa-miR-483-5p | miR-483-5p | -2.936 | 0.0000433 |
| hsa-miR-335-5p | miR-335-5p | -2.717 | 0.0004684 |
| hsa-miR-494-5p | miR-410-5p | -2.653 | 0.00000856 |
| hsa-miR-496 | miR-503-3p | -2.551 | 0.0003885 |
| hsa-miR-329-5p | miR-329-5p | -2.429 | 0.0007255 |
| hsa-miR-323b-3p | miR-323b-3p | -2.192 | 0.0008068 |
| hsa-miR-376c-3p | miR-376c-3p | -2.087 | 0.0001828 |
| hsa-miR-412-3p | miR-3551-5p | -1.911 | 0.000198967 |
| hsa-miR-1185-5p | miR-1185-5p | -1.796 | 0.002189283 |
| hsa-miR-323a-5p | miR-323-5p | -1.769 | 0.002138544 |
| hsa-miR-433-5p | miR-433-5p | -1.715 | 0.000110732 |
| hsa-miR-148a-5p | miR-148a-5p | -1.639 | 0.004385645 |
| hsa-miR-181c-3p | miR-181c-3p | -1.604 | 0.008470119 |
| hsa-miR-770-5p | miR-4712-5p | -1.552 | 0.000318609 |
| hsa-miR-148a-3p | miR-148a-3p | -1.522 | 0.022589679 |
| hsa-miR-432-3p | miR-432-3p | -1.501 | 0.004107626 |
| hsa-miR-656-3p | miR-656-3p | -1.492 | 0.000695908 |
| hsa-miR-6870-3p | miR-6870-3p | -1.484 | 0.0000772 |
| hsa-miR-181d-5p | miR-181a-5p | -1.461 | 0.018779829 |
| hsa-miR-376c-5p | miR-376b-5p | -1.449 | 0.000107946 |
| hsa-miR-381-5p | miR-381-5p | -1.438 | 0.000357403 |
| hsa-miR-889-5p | miR-889-5p | -1.38 | 0.004878617 |
| hsa-miR-380-3p | miR-380-3p | -1.368 | 0.000372672 |
| hsa-miR-181a-2-3p | miR-181a-2-3p | -1.316 | 0.00994975 |
| hsa-miR-3200-3p | miR-3200-3p | -1.259 | 0.00063732 |
| hsa-let-7e-5p | let-7a-5p | -1.224 | 0.000929496 |
| hsa-miR-424-3p | miR-424-3p | -1.195 | 0.02162024 |
| hsa-miR-24-1-5p | miR-24-1-5p | -1.189 | 0.005427255 |
| hsa-miR-134-3p | miR-12194-3p | -1.187 | 0.001928839 |
| hsa-miR-450b-5p | miR-450b-5p | -1.147 | 0.001508569 |
| hsa-miR-6760-3p | miR-6760-3p | -1.129 | 0.008689164 |
| hsa-miR-183-5p | miR-183-5p | -1.115 | 0.001589619 |
| hsa-miR-6783-5p | miR-6783-5p | -1.106 | 0.005410047 |
| hsa-miR-1-3p | miR-1-3p | -1.086 | 0.009412185 |
| hsa-let-7e-3p | let-7e-3p | -1.06 | 0.003062868 |
| hsa-miR-429 | miR-200b-3p | -1.043 | 0.001909078 |
| hsa-miR-891b | miR-891b | -1.034 | 0.008216114 |
| hsa-miR-542-3p | miR-542-3p | -1.034 | 0.004641399 |
| hsa-miR-1226-3p | miR-1226-3p | -1.021 | 0.019970605 |
| hsa-miR-200a-5p | miR-200a-5p | -1.017 | 0.006945214 |
| hsa-miR-96-5p | miR-96-5p | -1.003 | 0.018965945 |
