# Supplementary material for: iPSC-Derived Pancreatic Progenitors Lacking FOXA2 Reveal Alterations in miRNA Expression Targeting Key Pancreatic Genes
Source: Stem Cell Rev Rep. 2023 Feb 7;19(4):1082–97. doi: 10.1007/s12015-023-10515-3 (PMC10185633; doi:10.1007/s12015-023-10515-3)
Supplement: Supplementary file 7 — (PDF 513 KB) [file 12015_2023_10515_MOESM7_ESM.pdf]

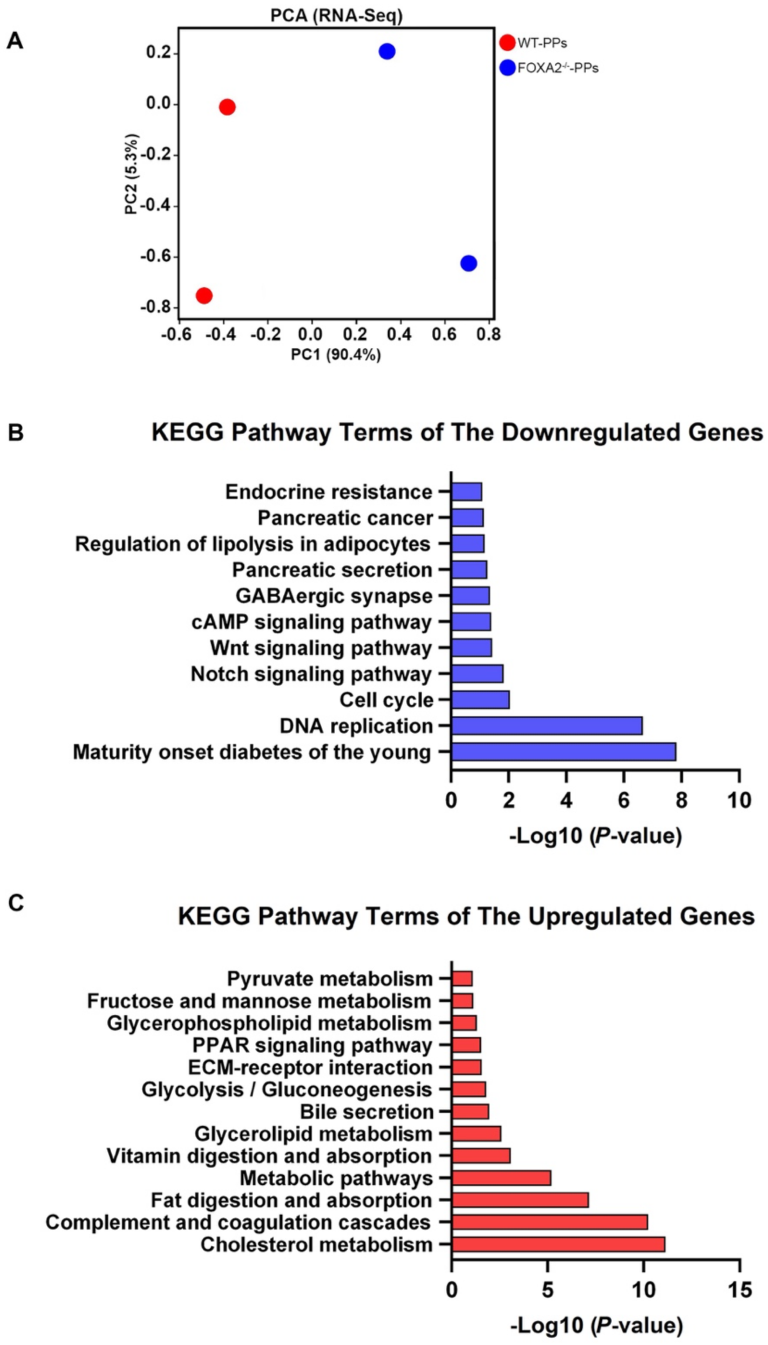

Supplementary Fig. 1. RNA-Seq analysis of differentially expressed mRNAs. (A) Principle component analysis of differentially expressed genes (DEGs) from RNA-seq of WT-PPs and *FOXA2*$^{-/-}$ PPs. **(B)** Analysis for all differentially expressed miRNAs (DEmiRs) from miRNA-seq of WT-PPs and *FOXA2*$^{-/-}$ PPs. KEGG pathway enrichment analysis of differentially expressed genes (DEGs) showing the associated enriched pathways in downregulated DEGs **(B)** and upregulated DEGs **(C)** in *FOXA2*$^{-/-}$ PPs using the DAVID function annotation tool. Enriched pathways were plotted against -Log10 (*P*-value).
