# Supplementary material for: iPSC-Derived Pancreatic Progenitors Lacking FOXA2 Reveal Alterations in miRNA Expression Targeting Key Pancreatic Genes
Source: Stem Cell Rev Rep. 2023 Feb 7;19(4):1082–97. doi: 10.1007/s12015-023-10515-3 (PMC10185633; doi:10.1007/s12015-023-10515-3)
Supplement: Supplementary file 8 — (PDF 446 KB) [file 12015_2023_10515_MOESM8_ESM.pdf]

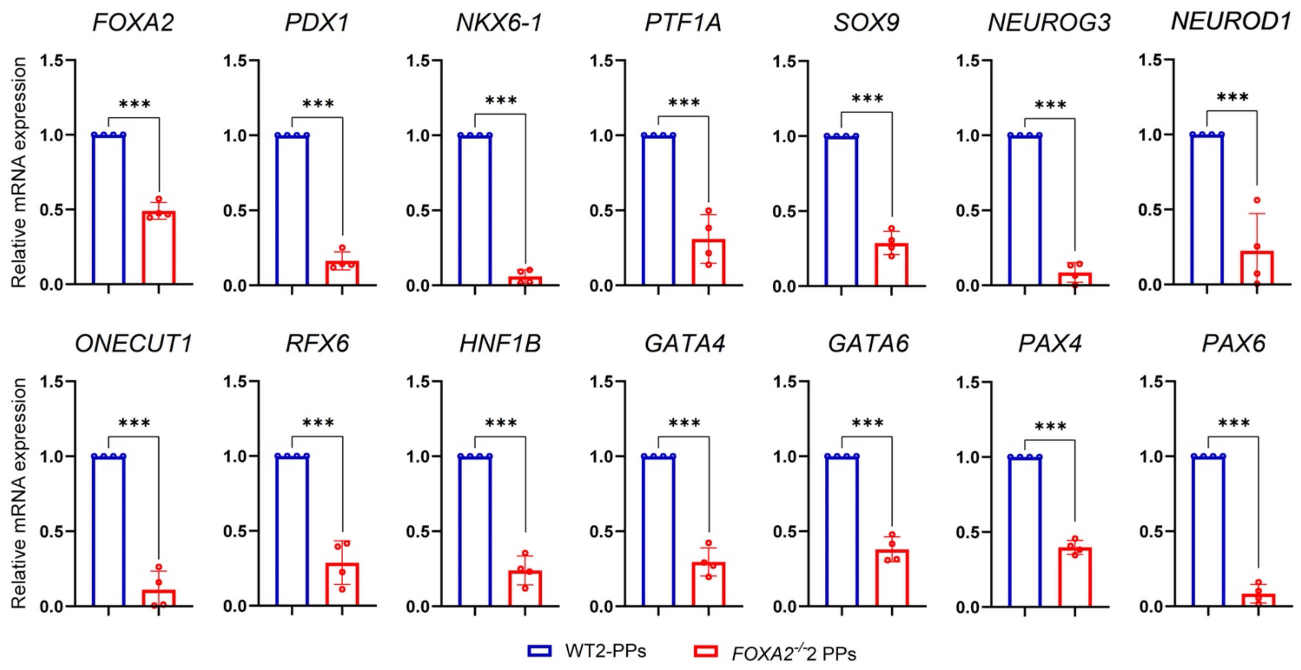


**Supplementary Fig. 2. RT-qPCR validation of selected key pancreatic markers from *FOXA2*$^{-/-}$ iPSC2 derived pancreatic progenitors (PPs).** Data are represented as mean ± SD; ***$p < 0.001$ (n=4).
