# Supplementary material for: iPSC-Derived Pancreatic Progenitors Lacking FOXA2 Reveal Alterations in miRNA Expression Targeting Key Pancreatic Genes
Source: Stem Cell Rev Rep. 2023 Feb 7;19(4):1082–97. doi: 10.1007/s12015-023-10515-3 (PMC10185633; doi:10.1007/s12015-023-10515-3)
Supplement: Supplementary file 9 — (PDF 189 KB) [file 12015_2023_10515_MOESM9_ESM.pdf]

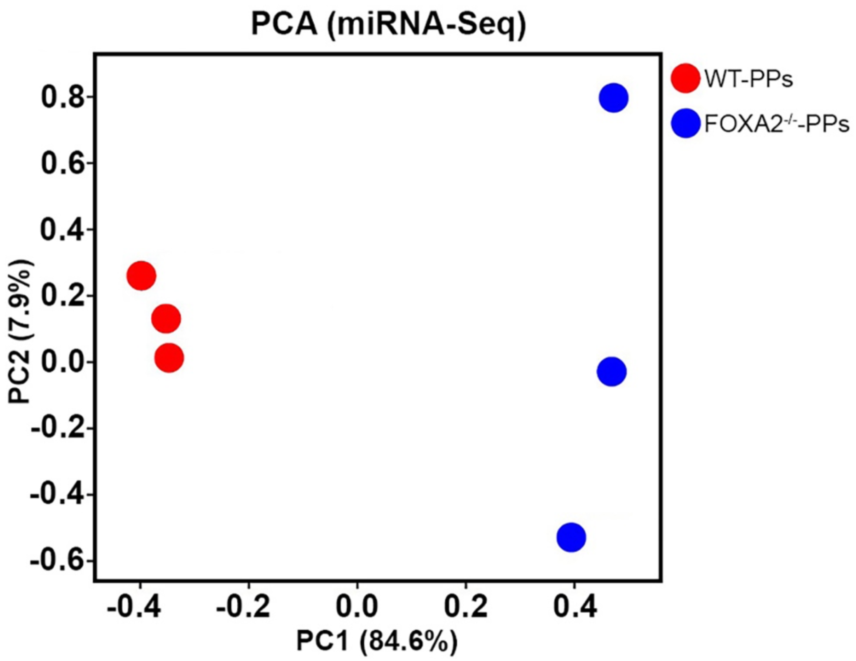

Supplementary Fig. 3. Principle component analysis of differentially expressed miRNAs. Analysis for all differentially expressed miRNAs (DEmiRs) from miRNA-seq of WT-PPs and *FOXA2*$^{-/-}$ PPs.
